# Snpdat: Easy and rapid annotation of results from *de novo* snp discovery projects for model and non-model organisms

**DOI:** 10.1186/1471-2105-14-45

**Published:** 2013-02-08

**Authors:** Anthony G Doran, Christopher J Creevey

**Affiliations:** 1 Teagasc Animal and Bioscience Research Department, Animal & Grassland Research and Innovation Centre, Teagasc, Grange, Dunsany, Co, Meath, Ireland; 2 Molecular Evolution and Bioinformatics Unit, Biology Department, NUI Maynooth, Maynooth, Co, Kildare, Ireland

**Keywords:** SNPs, Annotation, Software, Non-model organisms

## Abstract

**Background:**

Single nucleotide polymorphisms (SNPs) are the most abundant genetic variant found in vertebrates and invertebrates. SNP discovery has become a highly automated, robust and relatively inexpensive process allowing the identification of many thousands of mutations for model and non-model organisms. Annotating large numbers of SNPs can be a difficult and complex process. Many tools available are optimised for use with organisms densely sampled for SNPs, such as humans. There are currently few tools available that are species non-specific or support non-model organism data.

**Results:**

Here we present SNPdat, a high throughput analysis tool that can provide a comprehensive annotation of both novel and known SNPs for any organism with a draft sequence and annotation. Using a dataset of 4,566 SNPs identified in cattle using high-throughput DNA sequencing we demonstrate the annotations performed and the statistics that can be generated by SNPdat.

**Conclusions:**

SNPdat provides users with a simple tool for annotation of genomes that are either not supported by other tools or have a small number of annotated SNPs available. SNPdat can also be used to analyse datasets from organisms which are densely sampled for SNPs. As a command line tool it can easily be incorporated into existing SNP discovery pipelines and fills a niche for analyses involving non-model organisms that are not supported by many available SNP annotation tools. SNPdat will be of great interest to scientists involved in SNP discovery and analysis projects, particularly those with limited bioinformatics experience.

## Background

Single nucleotide polymorphisms (SNPs) are the most common genetic variant found in vertebrates and invertebrates [1]. SNPs are regularly utilised as the favoured molecular marker in association studies [2], genetic mapping [3] and population genetics [4]. Improving technologies and decreasing costs have enabled researchers to identify thousands of mutations, including rare variants, with potential influence on phenotypic variation [5,6]. More frequently non-bioinformatics researchers are required to perform analysis of increasingly large datasets. Disease susceptibility, agriculture and evolution are among the areas concerned with understanding the influence SNPs have on biological function and phenotypic variation of complex traits [7-9]. However, annotating large numbers of SNPs with this type of information can prove daunting and impractical to perform manually.

A number of bioinformatics tools for SNP annotation already exist (SNPit [10], SNPnexus [11], Snap [12], SNP Function Portal [13], SNPper [14], Fans [15], FunctSNP [16], Annovar [17]). Although there are over 50 reference sequences for eukaryotic species available from Ensembl (release 65) [18], there are currently only a small number of tools that enable analysis of non-human SNP data (e.g. Snat, Fans, FunctSNP, Annovar). Many tools that are more general can only analyse species with SNP information in dbSNP and some require that the SNPs being annotated already exist in dbSNP. Several tools try to circumvent this problem by returning information for known SNPs surrounding the unknown which works well for densely sampled species like humans but is not a viable option for almost all other species (Table 1).

**Table 1:** The number of SNP annotations (ss#) in dbSNP for species with a reference sequence available from ensembl and at least one SNP annotation in dbSNP (build 137)

**Species**	**Annotations in dbSNP**
*Homo sapiens* (Human)	60480978
*Mus musculus* (Mouse)	15721131
*Pongo abelii* (Orangutan)	10016093
*Bos taurus* (Cow)	9587248
*Rattus norvegicus* (Rat)	5227114
*Canis familiaris* (Dog)	3328578
*Gallus gallus* (Chicken)	3295452
*Macaca mulatta* (Macaque)	3041918
*Taeniopygia guttata* (Zebra Finch)	1751345
*Pan troglodytes* (Chimpanzee)	1660250
*Danio rerio* (Zebrafish)	1441888
*Ornithorhynchus anatinus* (Platypus)	1319269
*Monodelphis domestica* (Opossum)	1194131
*Equus caballus* (Horse)	1163580
*Tetraodon nigroviridis* (Tetraodon)	903110
*Sus scrofa* (Pig)	566003
*Felis catus* (Cat)	327037
*Caenorhabditis elegans* (C.elegans)	331438
*Meleagris gallopavo* (Turkey)	9256
*Gadus morhua* (Cod)	2140
*Gasterosteus aculeatus* (Stickleback)	1644
*Callithrix jacchus* (Marmoset)	10
*Gorilla gorilla* (Gorilla)	5

We have developed a simple to use SNP data analysis tool (SNPdat) specifically for use with organisms which are not supported by other tools and may have a small number of annotated SNPs available, but can equally be used to analyse datasets from organisms which are densely sampled for SNPs.

## Implementation

SNPdat is a cross-platform command line tool written in Perl, allowing easy incorporation into existing SNP discovery or annotation pipelines or even run by a user on a standard desktop machine. SNPdat can provide comprehensive annotation of both novel and known SNPs for any organism with a draft sequence and annotation.

Many available tools require the user to create a local database before SNP annotation can be performed (FunctSNP, Snat, Annovar, SNPper). However, this process is not practical in all cases or straightforward enough for inexperienced users. For example to perform SNP annotation using FunctSNP, users must first supply a list of Uniform Resource Locators (URLs) linked with online resource data files and then download them. They must then decompress any of these files matching specific suffixes, convert the data to SQL format to be imported to a SQLite database and finally import these files into the SQLite database. This is time-consuming and difficult for users inexperienced in bioinformatics to annotate even one SNP.

Additionally, some tools (Annovar, Snat) involve a number of pre-processing steps to parse and reformat either sequence or annotation files. This can be a difficult and confusing step for novice users, especially when dealing with non-model organisms. SNPdat does not require the creation of any local relational databases or pre-processing of any mandatory input files.

SNPdat requires only three input files; a variant calling formatted (VCF) file or a simple tab delimited text file (containing chromosome ID, genomic location and the mutation for each SNP to be analysed) as the SNP input file, a reference FASTA formatted sequence file for the species of interest, and a gene annotation file in GFF/GTF format. GTF files are a standard format for storing information on gene structure ( http://genome.ucsc.edu/FAQ/FAQformat.html#format4). GTF files define genomic structures as features. Typical features include coding sequences (CDS), exons, start and stop codons. Additional features may include untranslated regions (UTRs), introns and microRNAs.

Both FASTA and GTF files are available from Ensembl for over 50 eukaryotic species http://www.ensembl.org/info/data/ftp/index.html). Optional files include a processed file of SNP information from other databases such as dbSNP. SNPdat uses the extra information provided by this file to cross reference *de novo* SNPs against known annotations. Separate scripts are provided to automate the retrieval and format the data for any organisms with SNP information in dbSNP. Additional scripts which automate the retrieval of GTF, FASTA and dbSNP information are described in the following sections and are available from the SNPdat webpage ( http://code.google.com/p/snpdat/).

### Retrieval of GTF and FASTA information

**Figure 1:**
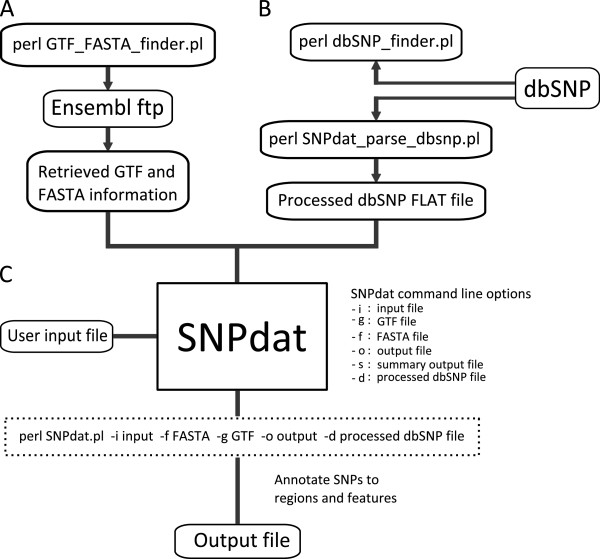
**Overview for using SNPdat and additional scripts available.** (**A**) Retrieval of GTF and FASTA information using GTF_FASTA_finder.pl. (**B**) Retrieval processing of data from dbSNP using dbSNP_finder.pl and SNPdat_parse_dbSNP.pl. (**C**) Command line options used to specify input/output files for SNPdat.

An additional script (GTF_FASTA_finder.pl) is provided to retrieve FASTA and GTF information for any of the organisms in Ensembl (Figure 1A). This is written in Perl but uses the system call cURL to retrieve the information from Ensembl. This script requires an internet connection. cURL is a part of most Linux distributions and Mac OS X and can also be provided for windows through cygwin, which is a collection of tools that provide a Linux-like environment for windows. This script is interactive; when run it prompts the user to select a release of Ensembl followed by an organism in that release. The GTF and FASTA files for that organism will be downloaded to the directory from which the script is run. Alternatively, GTF and FASTA information can be retrieved manually via the Ensembl website. SNPdat also works with genomic annotations from sources other than Ensembl as long as they are provided in GTF format. This includes the results of computationally derived annotations of *de novo* genomic assemblies, or transcriptomes.

### Retrieval of information from external databases

The script “dbSNP_finder.pl” retrieves SNP information for any organism in the dbSNP database (Figure 1B). This script also uses the cURL system call and requires a connection to the internet. Once run, the user is prompted to select an organism from all those currently with SNP information in dbSNP. The SNP information is then retrieved for that organism. SNP information from dbSNP can also be downloaded manually from the dbSNP ftp site ( ftp://ftp.ncbi.nih.gov/snp/organisms/). When dbSNP information has been retrieved, an additional script (SNPdat_parse_dbsnp.pl) can be used to convert the dbSNP file into a format suitable for use with SNPdat.

Conversion tools for databases that are not currently supported are available upon request.

### Running SNPdat

To run SNPdat, the user specifies the input/output files and desired options with a single command (Figure 1C). In the case of malformed commands, SNPdat will print an error message to the screen and a short example of how the correct command should look. SNPdat does not require the user to install any additional packages or modules and only uses modules included in the core installation of Perl.

Initially SNPdat reads the annotation information into memory from the GTF file. Each SNP is checked for errors such as non-numeric SNP locations and any warnings are printed to the output. All chromosome names provided by the user are compared against the annotation file. A warning message is printed to the output file for every SNP location provided which does not exist in the annotation. Once all SNPs have been parsed, SNPdat will read the FASTA file one chromosome at a time. To save on memory usage and time, any chromosomes that do not appear in the list of queried SNPs are skipped.

Output from SNPdat is presented in an easily accessible tab-delimited format containing up to 25 columns of information on each SNP queried. SNPdat returns information on genomic location of each SNP queried, including information on the distance to the nearest coding regions and other annotated sequence features, what those features are and where they start and finish (see Table 2 for more details). SNPdat contains algorithms for estimating information when not provided in either the genome file or the annotation file such as the total number of exons for each transcript containing a SNP, the estimated reading frame, (using the number of stop codons in each reading frame as a proxy), whether the region containing a SNP is exonic, intronic or intergenic and distances to coding regions for intronic and intergenic SNPs.

**Table 2:** Summary description of the annotations provided by SNPdat

**Column Number**	**Description**	**Example**
1	The queried SNPs chromosome ID	CHR25
2	The queried SNPs genomic location	286966
3	Whether or not the SNP was within a feature	Y
4	Region containing the SNP; either exonic, intronic, or intergenic	Exonic
5	Distance to nearest feature	NA
6	Either the closest feature to the SNP or the feature containing the SNP	CDS
7	The number of different features that the SNP is annotated to	2
8	The number of annotations of the current feature	[1/1]
9	Start of feature (bp)	286859
10	End of feature (bp)	287050
11	The gene ID for the current feature	ENSBTAG00000016571
12	The gene name for the current feature	ITFG3_BOVIN
13	The transcript ID for the current feature	ENSBTAT00000022045
14	The transcript name for the current feature	ITFG3_BOVIN
15	The exon that contains the current feature and the total number of annotated exons for the gene containing the feature	[3/11]
16	The strand sense of the feature	+
17	The annotated reading frame (when contained in GTF)	2
18	The reading frame estimated by SNPdat	NA
19	The estimated number of stop codons in the estimated reading frame	0
20	The codon containing the SNP, position in the codon and reference base and mutation	C[C/G]T
21	The amino acid for the reference codon and new amino acid with mutation in place	[P/R]
22	Whether or not the mutation is synonymous	N
23	The protein ID for the current feature	ENSBTAP00000022045
24	The RS identifier for queries that map to known SNPs	rs134558771
25	Error messages, warnings etc.	NA

SNPs that do not have sequence information in the FASTA file but have information in the GTF are still annotated by SNPdat. However, the returned information is limited to the first 17 columns and columns 23, 24 and 25 of the output file (Table 2).

### Non-coding SNPs

Next, all intronic and intergenic SNPs are identified and processed. The nearest feature to a non-coding SNP is identified and relevant data, such as distance to feature, feature IDs, strand sense, start and end position, is retrieved. If a SNP is equidistance from more than one feature, a separate line for each feature will be reported. Column seven in the output file contains the number of features reported for a SNP (see Table 2).

### Coding SNPs

All features that a SNP occurs in are identified and printed to separate lines. Information calculated and retrieved for a feature containing a SNP is contained in columns 9–17 of the output file (see Table 2). Columns 18–22 contain information estimated from the sequence of the feature such as the reading frame, the position in the codon, reference and mutant amino acid and whether or not the SNP is synonymous. The estimated reading frame is relative to the strand sense of the feature. If no strand sense is available from the GTF, SNPdat assumes that the strand sense is positive.

Finally, all SNPs are cross referenced against information retrieved from external databases such as dbSNP. SNPs that do not have sequence information in the FASTA file but have information in the GTF are still annotated by SNPdat. However, the returned information is limited to information which can be returned without reference to the DNA sequence (columns 1 to 17 and 23 to 25). See Table 2 for more details.

A tutorial demonstrating the use of SNPdat and the additional scripts is available from the SNPdat website ( http://code.google.com/p/snpdat/). A user manual and sample dataset are also available to download from here.

## Results and discussion

To demonstrate its ease of use, *de novo* SNPs discovered by Mullen *et al.* (2012) were annotated using SNPdat. As a comparison, Annovar was also used to analyse this dataset. This dataset consists of 4,566 SNPs discovered using high-throughput DNA sequencing of target-enriched pooled DNA samples of 83 genomic regions from groups of dairy cattle. The SNPs included novel and putative variants from 28 chromosomes including the X chromosome.

For SNPdat: EnsGene annotation and FASTA sequence files for *Bos taurus* were retrieved from the UCSC ftp site ( ftp://hgdownload.cse.ucsc.edu/goldenPath/bosTau4/). A GTF version of the ensGene annotation file was supplied to SNPdat along with the FASTA file. SNPdat does not require any pre-processing steps and so both these files were used as input for the software.

For Annovar: The same annotation and FASTA files were retrieved for use with Annovar. The FASTA file was pre-processed to create a sequence file using information from both the FASTA file and the ensGene annotation file. The new sequence file and original ensGene file were then supplied as input for Annovar.

Both tools annotate SNPs to coding regions (CDS), 3 prime untranslated regions (UTR), 5 prime UTR, intronic and intergenic regions (Table 3). SNPdat annotated SNPs to a larger number of features and transcripts (11,987 known features). Both tools identified mutations leading to stop gains, stop losses and other non-synonymous changes.

**Table 3:** The number of SNPs annotated to different regions by SNPdat and Annovar

**Region**	**SNPdat**	**Annovar**
Coding	299	299
3 prime UTR	108	105
5 prime UTR	29	28
Intronic	3285	3284
Intergenic	845	845
Misc.	0	5
Total	4566	4566

Misc features include non-coding RNA and splicing. These features were not included in the GTF version of the ensGene annotation file and so SNPdat was unable to identify them as such.

**Figure 2:**
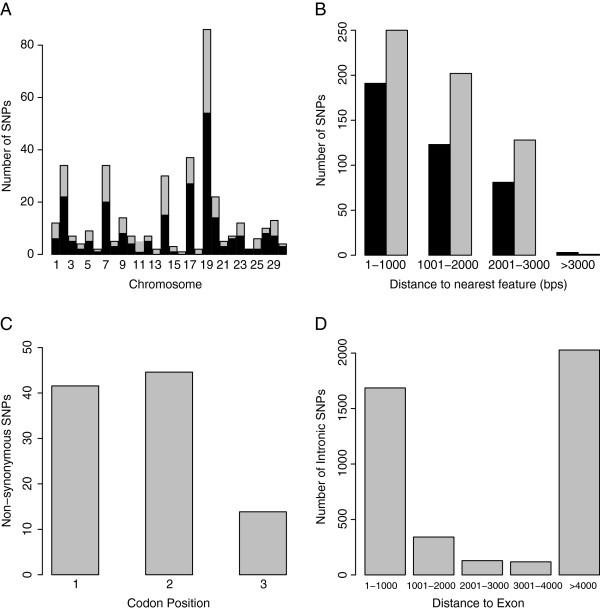
**Sample of plots obtained using the results of SNPdat.** (**A**) The number of non-synonymous (black) and total number of exonic SNPs (grey) found on each chromosome. (**B**) Distances of intergenic SNPs, upstream (black) and downstream (grey) to the nearest transcripts. (**C**) Synonymous versus non-synonymous SNPs: 231 exonic SNPs were non-synonymous. 96 (41.56%) in the first codon position, 103 (44.59%) in the second codon position and 32 (13.85%) in the third codon position. (**D**) Distances of Intronic SNPs to the nearest exon.

Both Annovar and SNPdat annotated 299 SNPs in coding regions to a total of 382 transcripts. Of these, 231 SNPs were non-synonymous and 151 SNPs were synonymous mutations (Figure 2A). From the SNPdat output file it was possible to determine upstream and downstream distances for SNPs to coding regions (Figure 2B). Also, from the SNPdat output file it was determined that 96, 103 and 32 non-synonymous SNPs occurred in the first, second and third codon position respectively (Figure 2C).

SNPdat and Annovar both found a large proportion of (77%) of intergenic SNPs within 2,000 base pairs of coding regions. Additionally, from SNPdat output file it was determined that 39% of intronic SNPs were within a 1,000 base pair region surrounding exons (Figure 2D).

## Conclusion

The rationale behind SNPdat is to provide a simple to use tool for researchers annotating the results of *de novo* SNP discovery projects. It is especially intended for use by researchers with limited bioinformatic experience. It can provide a valuable insight into the functional roles associated with discovered SNPs and cross reference information with external sources. As a command line tool it can easily be incorporated into existing SNP discovery pipelines and fills a niche for analyses involving non-model organisms that are not supported by many available SNP annotation tools.

## Availability and requirements

**Project name**: SNPdat

**Project home page**: http://code.google.com/p/snpdat

**Operating system**: Platform independent

**Programming language**: Perl

**Other requirements**: Perl

**Licence**: GPLv2

**Any restrictions to use by non-academics**: None

## Abbreviations

SNP: Single Nucleotide Polymorphism; URL: Uniform Resource Locator; VCF: Variant Calling Format; GTF: Gene Transfer Format.

## Competing interests

The authors declare that they have no competing interests.

## Authors’ contributions

CJC conceived the project. CJC and AGD designed and tested algorithms. AGD wrote the code for SNPdat. Both authors read and approved the final manuscript.

## References

[B1] Cohuet A Krishnakumar S Simard F Morlais I Koutsos A Fontenille D Mindrinos M Kafatos FC SNP discovery and molecular evolution in Anopheles gambiae, with special emphasis on innate immune system BMC Genomics 2008 9 227 10.1186/1471-2164-9-227 18489733 PMC2405807

[B2] WTCCC Genome-wide association study of 14,000 cases of seven common diseases and 3,000 shared controls Nature 2007 447 7145 661 678 10.1038/nature05911 17554300 PMC2719288

[B3] Hoskins RA Phan AC Naeemuddin M Mapa FA Ruddy DA Ryan JJ Young LM Wells T Kopczynski C Ellis MC Single nucleotide polymorphism markers for genetic mapping in Drosophila melanogaster Genome Res 2001 11 6 1100 1113 10.1101/gr.GR-1780R 11381036 PMC311062

[B4] Tishkoff SA Verrelli BC Patterns of human genetic diversity: implications for human evolutionary history and disease Annu Rev Genomics Hum Genet 2003 4 293 340 10.1146/annurev.genom.4.070802.110226 14527305

[B5] Altshuler D Pollara VJ Cowles CR Van Etten WJ Baldwin J Linton L Lander ES An SNP map of the human genome generated by reduced representation shotgun sequencing Nature 2000 407 6803 513 516 10.1038/35035083 11029002

[B6] Mullen MP Creevey CJ Berry DP McCabe MS Magee DA Howard DJ Killeen AP Park SD McGettigan PA Lucy MC Polymorphism discovery and allele frequency estimation using high-throughput DNA sequencing of target-enriched pooled DNA samples BMC Genomics 2012 13 1 16 10.1186/1471-2164-13-16 22235840 PMC3315736

[B7] Allan MF Smith TP Present and future applications of DNA technologies to improve beef production Meat Sci 2008 80 1 79 85 10.1016/j.meatsci.2008.05.023 22063172

[B8] Corona E Dudley JT Butte AJ Extreme evolutionary disparities seen in positive selection across seven complex diseases PLoS One 2010 5 8 e12236 10.1371/journal.pone.0012236 20808933 PMC2923198

[B9] Cutter AD Choi JY Natural selection shapes nucleotide polymorphism across the genome of the nematode Caenorhabditis briggsae Genome Res 2010 20 8 1103 1111 10.1101/gr.104331.109 20508143 PMC2909573

[B10] Shen TH Carlson CS Tarczy-Hornoch P SNPit: a federated data integration system for the purpose of functional SNP annotation Comput Methods Programs Biomed 2009 95 2 181 189 10.1016/j.cmpb.2009.02.010 19327864 PMC2680224

[B11] Chelala C Khan A Lemoine NR SNPnexus: a web database for functional annotation of newly discovered and public domain single nucleotide polymorphisms Bioinformatics 2009 25 5 655 661 10.1093/bioinformatics/btn653 19098027 PMC2647830

[B12] Li S Ma L Li H Vang S Hu Y Bolund L Wang J Snap: an integrated SNP annotation platform Nucleic Acids Res 2007 35 D707 710 Database issue) 10.1093/nar/gkl969 17135198 PMC1751554

[B13] Wang P Dai M Xuan W McEachin RC Jackson AU Scott LJ Athey B Watson SJ Meng F SNP Function Portal: a web database for exploring the function implication of SNP alleles Bioinformatics 2006 22 14 e523 529 10.1093/bioinformatics/btl241 16873516

[B14] Riva A Kohane IS SNPper: retrieval and analysis of human SNPs Bioinformatics 2002 18 12 1681 1685 10.1093/bioinformatics/18.12.1681 12490454

[B15] Liu CK Chen YH Tang CY Chang SC Lin YJ Tsai MF Chen YT Yao A Functional analysis of novel SNPs and mutations in human and mouse genomes BMC Bioinforma 2008 9 12 S10 10.1186/1471-2105-9-S12-S10 PMC2638150 19091009

[B16] Goodswen SJ Gondro C Watson-Haigh NS Kadarmideen HN FunctSNP: an R package to link SNPs to functional knowledge and dbAutoMaker: a suite of Perl scripts to build SNP databases BMC Bioinforma 2010 11 311 10.1186/1471-2105-11-311 PMC2901372 20534127

[B17] Wang K Li M Hakonarson H ANNOVAR: functional annotation of genetic variants from high-throughput sequencing data Nucleic Acids Res 2010 38 16 e164 10.1093/nar/gkq603 20601685 PMC2938201

[B18] Flicek P Amode MR Barrell D Beal K Brent S Carvalho-Silva D Clapham P Coates G Fairley S Fitzgerald S Ensembl 2012 Nucleic Acids Res 2012 40 D84 90 Database issue 10.1093/nar/gkr991 22086963 PMC3245178

